# Cas9 Nickase-Assisted RNA Repression Enables Stable and Efficient Manipulation of Essential Metabolic Genes in *Clostridium cellulolyticum*

**DOI:** 10.3389/fmicb.2017.01744

**Published:** 2017-09-07

**Authors:** Tao Xu, Yongchao Li, Zhili He, Joy D. Van Nostrand, Jizhong Zhou

**Affiliations:** ^1^Institute for Environmental Genomics and Department of Microbiology and Plant Biology, University of Oklahoma, Norman OK, United States; ^2^Earth Sciences Division, Lawrence Berkeley National Laboratory, Berkeley CA, United States; ^3^State Key Joint Laboratory of Environment Simulation and Pollution Control, School of Environment, Tsinghua University Beijing, China

**Keywords:** essential genes, genome editing, gene repression, metabolic engineering, consolidated bioprocessing, C*lostridium cellulolyticum*

## Abstract

Essential gene functions remain largely underexplored in bacteria. *Clostridium cellulolyticum* is a promising candidate for consolidated bioprocessing; however, its genetic manipulation to reduce the formation of less-valuable acetate is technically challenging due to the essentiality of acetate-producing genes. Here we developed a Cas9 nickase-assisted chromosome-based RNA repression to stably manipulate essential genes in *C. cellulolyticum*. Our plasmid-based expression of antisense RNA (asRNA) molecules targeting the phosphotransacetylase (*pta*) gene successfully reduced the enzymatic activity by 35% in cellobiose-grown cells, metabolically decreased the acetate titer by 15 and 52% in wildtype transformants on cellulose and xylan, respectively. To control both acetate and lactate simultaneously, we transformed the repression plasmid into lactate production-deficient mutant and found the plasmid delivery reduced acetate titer by more than 33%, concomitant with negligible lactate formation. The strains with *pta* gene repression generally diverted more carbon into ethanol. However, further testing on chromosomal integrants that were created by double-crossover recombination exhibited only very weak repression because DNA integration dramatically lessened gene dosage. With the design of a tandem repetitive promoter-driven asRNA module and the use of a new Cas9 nickase genome editing tool, a chromosomal integrant (LM3P) was generated in a single step and successfully enhanced RNA repression, with a 27% decrease in acetate titer on cellulose in antibiotic-free medium. These results indicate the effectiveness of tandem promoter-driven RNA repression modules in promoting gene repression in chromosomal integrants. Our combinatorial method using a Cas9 nickase genome editing tool to integrate the gene repression module demonstrates easy-to-use and high-efficiency advantages, paving the way for stably manipulating genes, even essential ones, for functional characterization and microbial engineering.

## Introduction

Essential genes are indispensable for building up the chassis of living organisms (Glass et al., 2006), and accounts for 5–80% of bacterial genomes (Gao and Zhang, 2011). Investigation into these genes will provide insights on basic biological functions and allow for the discovery of cellular activities with industrial or biomedical potentials (Lee et al., 2009; Juhas et al., 2012). Since genetic knock-outs of essential genes are lethal and then unobtainable (Glass et al., 2006), knock-down strategies are applicable and widely used (Ji et al., 1999). There are three major approaches available for targeted gene repression in bacteria, including antisense RNA (asRNA)-mediated repression (Desai and Papoutsakis, 1999; Perret et al., 2004; Thomason and Storz, 2010), Hfp-dependent RNA repression (Man et al., 2011; Na et al., 2013) and nuclease-null Cas9-mediated repression (which is named CRISPRi) (Bikard et al., 2013; Qi et al., 2013). The latter two require an RNA binding protein, Hfp chaperone and non-catalytic Cas9 endonuclease, respectively, which need to be consistently co-expressed with RNA molecules that recognize target transcripts. Plasmid-based expression of these components has been widely applied in bacteria (Desai and Papoutsakis, 1999; Perret et al., 2004; Thomason and Storz, 2010; Man et al., 2011; Bikard et al., 2013; Na et al., 2013); however, concerns are raised about the stability and antibiotic dependence of plasmid-based expression (Lee et al., 1993; Friehs, 2004), especially in industrial microorganisms, and potential side effects caused by the specificity of RNA binding proteins (Martinez-Alonso et al., 2010; Bikard et al., 2013; Qi et al., 2013). Development of a relatively clean, easy and efficient approach allowing for rapidly generating stable knock-down mutants would increase our ability to study and manipulate essential genes. Considering the easy-to-use and highly efficient advantages of CRISPR/Cas9-based genome editing tools (Xu et al., 2014, 2015) and the simplicity and universality of antisense RNA-mediated repression (Thomason and Storz, 2010), here we propose a combination of these two methods using Cas9 technology to integrate antisense RNA modules into the genome. By doing so, knock-down mutants can be created in a single step with features that are plasmid-independent and can be sustained without using antibiotics.

*Clostridium cellulolyticum* H10, a model organism of mesophilic cellulolytic Clostridia, is an excellent consolidated bioprocessing host (Desvaux, 2005; Lynd et al., 2005). It can hydrolyze lignocellulose without adding commercial cellulases and simultaneously ferment a variety of C5 and C6 sugars to end products (lactate, acetate and ethanol) (Desvaux, 2005). Metabolic engineering significantly improved microbial characteristics via overexpressing foreign genes of intended pathways (Guedon et al., 2002; Higashide et al., 2011; Li et al., 2014; Lin et al., 2015), or eliminating competing and promiscuous pathways (Shaw et al., 2008; Li et al., 2012; Papanek et al., 2015). In *C. cellulolyticum*, a double mutation of lactate and malate dehydrogenase genes (Δ*ldh Δmdh*, hereafter LM mutant) abolished lactate production, accompanied with carbon flux redistribution (Li et al., 2012). However, no knock-out mutants of acetate producing genes, phosphotransacetylase (*pta*) and acetate kinase (*ack*), were isolated to abolish acetate formation, suggesting that these two genes are essential in *C. cellulolyticum* under the condition tested (Li et al., 2012). The difficulty hampered combined metabolic engineering to maximize the elimination of less useful products (acetate and lactate) as demonstrated in the triple mutant of *T. saccharolyticum* (Δ*ldh* Δ*pta* Δ*ack*) (Shaw et al., 2008) and the quintuple mutant of *C. thermocellum* (Δ*hpt*, Δ*ldh*, Δ*hydG*, Δ*pfl*, and Δ*pta-ack*) (Papanek et al., 2015). With the aim of reducing acetate formation by manipulating these essential metabolic genes, both the traditional double-crossover recombination (Heap et al., 2012) and the newly developed Cas9 nickase-triggered homologous recombination, which has been proven in *C. cellulolyticum* (Xu et al., 2015), were employed to deliver the cassettes of antisense RNA expressing modules into a targeted genomic locus. The RNA repression effect in plasmid transformants and chromosomal integrants was determined and compared. Then, we improved the repression effect in chromosomal integrants by using a synthetic tandem promoter. The genetic regulatory strategies established in this study will greatly expand our ability to stably tune the expression of genes for genetic and metabolic engineering of bacteria.

## Materials and Methods

### Plasmid Construction

To construct plasmids expressing asRNAs, a partial transcriptional region of either the *pta* or *ack* gene, spanning from the predicted transcriptional start site to the downstream site approximately 120 bp away from the start codon, was amplified with specific primer sets (Supplementary Table S1). The exact size of asRNAs varied depending on primers used to generate PCR amplicon with the size of >100 bp which can trigger efficient RNA repression in bacteria (Perret et al., 2004; Wang and Kuramitsu, 2005). Then, qualified PCR products were fused with the *Clostridium pasteurianum* ferredoxin promoter in an inverted orientation by assembling with BamHI-linearized pRNAi control plasmid (Gibson assembly kit, NEB), generating pRNAi-pta and pRNAi-ack harboring Fd::pta asRNA module and Fd::ack asRNA module respectively.

To conduct chromosomal integration of asRNA modules via double-crossover recombination (Heap et al., 2012), plasmids containing these asRNA modules flanked by homologous arms were constructed as follows. First, DNA fragments of interest were amplified and purified separately: promoterless *mlsR* gene amplified from pLyc1217Er (Li et al., 2012); asRNA cassettes from pRNAi and pRNAi-pta; upper and lower homologous arms from the wildtype (WT) genome; and linear backbone from pRNAi. These fragments were then mixed and assembled together using a Gibson assembly kit and the resulting reaction product was transformed into *Escherichia coli* for colony screening. Consequently, plasmids pLyc045 and pLyc046 were constructed with 3198up-mlsR-empty asRNA-3198down and 3198up-mlsR-pta asRNA-3198down for the integration of Fd::empty and Fd::pta asRNA cassette at the selected locus. Similarly, to integrate the Fd::afp cassette there, pLyc048 was constructed with 3198up-mlsR-Fd::afp-3198down.

To increase asRNA expression, a tandem promoter cluster consisting of three P4 promoters was synthesized and then fused with the same asRNA region by overlapping PCR, generating a 3P4::pta asRNA cassette. Since Cas9 nickase-based chromosomal integration is simpler and much more efficient (Xu et al., 2015), it was applied to deliver 3P4::pta asRNA into the genome. The 23-bp target site (5^′^-AAGTAAGAAACATTTGGTTCCGG-3^′^) was located in the downstream intergenic region of Ccel_3198. pCas9n-3198D with a customized donor was constructed in two steps. First, pCas9n-3198D reported previously was linearized by BamHI (Xu et al., 2015) and then assembled with both left and right homologous arms amplified from the WT genome, generating pCas9n-3198D with NcoI-containing donor. Second, the resulting plasmid was linearized by NcoI for the assembly with the 3P4::pta asRNA cassette, generating pCas9n-3198D-donor. Descriptions of all plasmids used in this study were listed in **Table 1**.

**Table 1:** Plasmids and strains used in this study.

Name	Description	Reference
**Strain**		
*E. coli* TOP10	Host cells for plasmid construction	Invitrogen
WT	Wildtype *C. cellulolyticum* H10	ATCC
WT-P	WT with pRNAi control plasmids	This study
WT-P-pta	WT with pRNAi-pta plasmids	This study
WT-P-ack	WT with pRNAi-ack plasmids	This study
WT-P-afp	WT with pFd-AFP plasmids	(Xu et al., 2015)
WT-G	WT with a chromosomal Fd::empty cassette	This study
WT-G-afp	WT with a chromosomal Fd::afp cassette	This study
LM	Δ*ldh*Δ*mdh*	(Li et al., 2012)
LM-P	LM with pRNAi plasmids	This study
LM-P-pta	LM with pRNAi-pta plasmids	This study
LM-G	LM with a chromosomal RNAi control	This study
LM-G-pta	LM with a chromosomal Fd::pta asRNA cassette	This study
**Plasmid**		
pRNAi	CMP^r^ in E. coli; TMP^r^ in H10; Fd::empty cassette	(Xu et al., 2015)
pRNAi-pta	pRNAi derivative with a Fd::pta asRNA cassette	This study
pRNAi-ack	pRNAi derivative with a Fd::ack asRNA cassette	This study
pLyc045	pRNAi derivative with 3198up-mlsR-Fd::empty-3198down	This study
pLyc046	pRNAi derivative with 3198up-mlsR-Fd::pta asRNA-3198down	This study
pFd-AFP	pRNAi derivative with a Fd::afp cassette	(Xu et al., 2015)
pLyc048	pRNAi derivative with 3198up-mlsR-Fd::afp-3198down	This study
pCas9n-3198D	pRNAi derivative with a cas9 nickase and a gRNA targeting the 3198D site	(Xu et al., 2015)
pCas9n-3198D with donor	pCas9n-3198D derivative with left arm-3P4::pta asRNA-right arm	This study

### Bacterial Strains and Culture Conditions

*Escherichia coli* Top10 (Invitrogen) was used for molecular cloning. Transformants were grown at 37°C in Luria-Bertani medium supplemented with kanamycin (50 μg/ml) or chloramphenicol (15 μg/ml) when required. *Clostridium cellulolyticum* H10 and developed strains were cultured anaerobically at 34°C in VM media supplemented with 2.0 g/L yeast extract and various carbon sources (Higashide et al., 2011). Transformants of H10 and LM mutant were selected by erythromycin (15 μg/ml) or thiamphenicol (15 μg/ml). Colonies of *C. cellulolyticum* strains were developed on solid VM plates containing 1% (w/v) agar, 5 g/L cellobiose and antibiotics. Plasmid transformants were generated by transforming the corresponding plasmids. Chromosomal integrants, WT-G and WT-G-afp, were generated by transforming WT with pLyc045 and pLyc048, respectively. Chromosomal integrants, LM-G, LM-G-PTA and LM3P, were generated by transforming the LM mutant with pLyc045, pLyc046, and pCas9n-3198D-donor, respectively. All constructed strains are listed in **Table 1**.

### *C. cellulolyticum* Transformation

*Clostridium cellulolyticum* electro-competent cells and methylated plasmids were prepared as previously described (Li et al., 2014). Briefly, *C. cellulolyticum* strains were grown at 34°C in liquid VM medium with 5 g/L cellobiose and 2 g/L yeast extract until reaching an OD_600_ = 0.3–0.5. The cell culture was then chilled on ice and then centrifuged at 4°C and 3,000 *g* for 8 min, and the cell pellets were washed at least three times with an equal volume of ice-cold anoxic electroporation buffer (270 mM sucrose, 1 mM MgCl_2_ and 5 mM sodium phosphate buffer, pH 7.4). Lastly, competent cells made from every 10 ml of cell culture were resuspended in 100 μl chilled electroporation buffer for further use. Plasmid DNA was methylated with MspI methyltransferase (NEB), followed by DNA purification and quantification. For each transformation, a 100 μl cell suspension was mixed with 2.0 μg of methylated plasmids and then electroporated in a 2-mm cuvette (1.25 kV, 5 ms, 1 square pulse) with a Gene Pulser Xcell (Bio-Rad) in the anaerobic chamber. After electroporation, cells were recovered for 12–24 h in antibiotic-free VM medium with 5 g/L cellobiose and 2 g/L yeast extract, and then selected by appropriate antibiotics on agar VM plates.

### Enzyme Activity Assay

To measure enzyme activities, cell-free extracts were made from cellobiose-grown *C. cellulolyticum* strains at the mid-log phase using CelLytic B cell lysis reagent (Sigma). Crude extracts were centrifuged at 14,000 *g* at 4°C for 10 min to remove insoluble cell debris. Then, the protein concentration was determined with a BCA assay kit (Thermo Scientific), using bovine serum albumin as a standard. Crude protein samples were stored on ice until assayed. One unit of activity is defined as the amount of enzyme that catalyzes the conversion of one micromole substrate per minute under the experimental conditions. The specific activity was defined as the units of enzyme activity per mg of total protein.

Acetate kinase activity was measured in the direction of acyl phosphate formation (Rose, 1955). The reaction was initiated by adding 0.4 μg of protein sample to 320 μl reaction mixture [200 mM Tris-HCl (pH 7.4), 10 mM ATP, 10 mM MgCl2, 6% (w/v) hydroxylamine hydrochloride (neutralized with KOH before addition), and 267 mM potassium acetate]. The reaction was incubated at 25°C for 10 min and stopped by adding 320 μl of 10% (w/v) ice-cold trichloroacetic acid. The experimental control was made with boiled protein samples in the above reaction mixture. Color was developed by adding 320 μl 2.5% (w/v) FeCl3 in 2.0 N HCl. The absorbance at 540 nm was measured with a Biowave II spectrophotometer (WPA). An extinction coefficient of 0.169/mM/cm was used to calculate the activity of acetate kinase.

Phosphotransacetylase activity was measured by monitoring the liberation of coenzyme A at 405 nm (Andersch et al., 1983). The reaction was initiated by adding 2 μg of cell-free extracts to 1 mL of reaction mixture [0.1 M potassium phosphate buffer (pH 7.4), 0.2 mM acetyl-CoA, 0.08 mM 5, 5^′^-dithio-bis (2-nitrobenzoate)] and then incubated at 25°C for 10 min. The experimental control was made with boiled protein samples in the above reaction mixture. The absorbance at 405 nm was measured with a Biowave II spectrophotometer (WPA). An extinction coefficient of 13.6/mM/cm was used to calculate phosphotransacetylase activity.

Aldehyde dehydrogenase activity was measured by monitoring NADH oxidation which decreases absorbance at 340 nm (Brown et al., 2011). Protein samples (10 μl) were added to 1 mL reaction mixture [100 mM Tris-HCl (pH 7.6), 1 mM DTT buffer, 5 μM FeSO_4_, 0.5 mM NADH, 55 mM acetaldehyde] and incubated at 34°C for 20 min before absorbance measurement. The experimental control was made with boiled protein samples in the above reaction mixture. An extinction coefficient of 6.22/mM/cm was used to calculate aldehyde dehydrogenase activity.

### Measurement of Cell Growth and Fermentation Products

*Clostridium cellulolyticum* strains were revived in VM media with 5 g/L cellobiose, and antibiotic was added if necessary. The cellobiose-grown cultures at an OD_600_ of 0.5–0.7 were used for 1% inoculation into 50 ml fresh VM media with 5 g/L cellobiose, 10 g/L Avicel PH101 crystalline cellulose (Sigma) or 10 g/L beach wood xylan (Sigma). Each strain had three biological replicates. Cell growth on cellobiose was profiled by monitoring OD600 with a spectrophotometer. When grown on cellulose and xylan, 1 mL of cell culture was sampled and then stored at -80°C for metabolite measurements.

Cell biomass on cellulose was estimated by total protein measurement. The cells were lysed with 0.2 N NaOH/1% w/v SDS solution for 60 min at 25°C, and then neutralized with 0.8 N HCl. After centrifugation at 12,000 *g* for 10 min, the supernatant was used for protein quantification with a BCA assay kit.

To measure fermentation products (including lactate, acetate, and ethanol), the fermentation broth was filtered through 0.2 μm filters, acidified with 0.025% H_2_SO_4_ and then subjected to high-performance liquid chromatography (HPLC) analyses with an Agilent 1200 system (Agilent Technologies) equipped with a variable-wavelength (190–600 nm) detector (with UV absorption measured at 245 nm) and an ion-exclusion column (Aminex HPX-87H; 300 mm × 7.8 mm; Bio-Rad Laboratories, Hercules, CA, United States). HPLC operating parameters included a column temperature at 65°C, 0.025% sulfuric acid as the mobile phase at a flow rate of 0.6 ml/min and 50 μl sample injected (Hemme et al., 2011). Referring to the corresponding standard curves, the concentration of each product was calculated.

### Quantitative Real-Time PCR

To compare the gene copy number and the transcript amount of *afp* gene in P-AFP transformant and G-AFP integrant, qRT-PCR was conducted as follows. Cell samples were collected from cellobiose-grown cultures at mid-log phase (OD_600_ = ∼0.45). To compare gene copy number, DNA was extracted by heating at 98°C for 6 min. Heat-treated samples were centrifuged to remove insoluble cell debris. Then, the supernatants were subjected to qRT-PCR analysis using iTaq SYBR Green Supermix with ROX (Bio-Rad) on a Bio-Rad iQ5 thermal cycler. The *recA* gene in the genome was used as an internal calibrator to determine the copy number of *afp* gene. Primers used in qRT-PCR are listed (Supplementary Table S1). Results were analyzed with the Pfaffl method (Pfaffl, 2001).

To compare the transcript amount of *afp* gene by qRT-PCR, cells were lysed by TRIzol Reagent (Invitrogen) followed by total RNA extraction and purification with NucleoSpin RNAII kit (Macherey-Nagel). SuperScript III Reverse Transcriptase (Invitrogen) was applied to convert RNA to cDNA by following the manufacturer’s protocol. cDNA products were diluted as appropriate and used as templates for qRT-PCR. Similarly, results were analyzed with the Pfaffl method using *recA* as the reference gene (Pfaffl, 2001).

### Microscopy and Flow Cytometry

Fluorescence intensity of the anaerobic fluorescence protein was evaluated by fluorescent microscopy and flow cytometry. *C. cellulolyticum* strains at the mid-log phase were harvested, washed twice with the anaerobic PBS buffer and then suspended in the same buffer before loading onto microscope slides. Slides were imaged using Olympus BX51 fluorescence microscope equipped with optical filter sets with excitation at 490 nm and emission at 525 nm for the green fluorescence. The images were collected by an Olympus DP71 digital camera.

Flow cytometry analysis was performed on a BD Accuri C6 flow cytometer (BD Biosciences) (Li et al., 2014). All samples were diluted with the anaerobic PBS buffer to similar concentrations, then run through the flow cytometer under aerobic condition following the manufacturer’s instructions. The run limit was set up as 10,000 events at a slow flow rate, the threshold as 40,000 on FSC-H. The fluorescence was detected with a FL1 detector with a 530/30 filter. The data were collected and analyzed with the CFlow software.

## Results and Discussion

### Plasmid-Based Antisense RNA Expression

**FIGURE 1:**
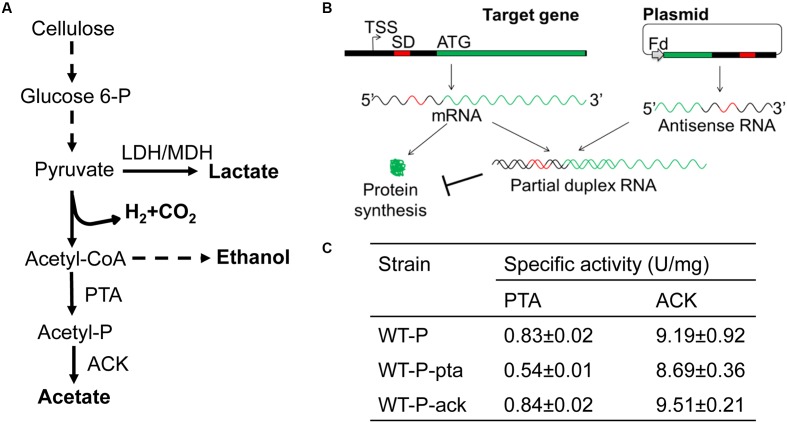
**(A)** Major metabolic pathways in *C. cellulolyticum*. Acetyl-CoA as a key intermediate metabolite, apart from being used to produce ethanol, can be converted to acetyl-phosphate by phosphotransacetylase (PTA, encoded by *pta* gene) and then to acetate by acetate kinase (ACK, encoded by *ack* gene). L-lactate dehydrogenase (LDH) and L-malate dehydrogenase (MDH) are functional in one-step lactate production from pyruvate. Dashed arrows refer to multiple enzymatic reactions. **(B)** Design of antisense RNAs (asRNAs) to repress *pta* and *ack* genes. For each target gene, the transcriptional region spanning from the predicted transcriptional start site (TSS) to the downstream site approximately 120-bp from the start codon (ATG), containing the Shine-Dalgarno sequence (SD), was amplified and reversely inserted downstream of the ferredoxin (Fd) promoter, generating the Fd::asRNA module. AsRNAs would interfere with the transcription, stability and translation of the target gene. **(C)** Enzyme assays of PTA and ACK in crude cell-free extracts. Mean and standard deviations of specific enzyme activities were calculated from three biological replicates.

To test the use of asRNA molecules to repress acetate production in *C. cellulolyticum*, we targeted *pta* encoding phosphotransacetylase (PTA) and *ack* encoding acetate kinase (ACK), both of which are key to produce acetate from acetyl-CoA (**Figure 1A**) and essential for cell survival (Li et al., 2012). For each target gene, its 5^′^ transcriptional region with a length of approximately 120 bp was inserted in a reverse orientation under the control of a ferredoxin promoter to produce asRNAs which will interfere with the stability and translation of target transcripts (**Figure 1B**) (Thomason and Storz, 2010). The empty asRNA plasmid (pRNAi), customized pRNAi-pta and pRNAi-ack plasmids targeting *pta* and *ack*, respectively, were constructed and transformed into WT, generating WT-P control, WT-P-pta and WT-P-ack transformants (where P means plasmid-based expression). Then, we examined the repression effect of designed asRNAs by measuring enzyme activities of PTA and ACK in these strains that were grown on 5 g/L cellobiose (**Figure 1C**). Our results showed that (i) PTA activity in WT-P-pta (0.54 ± 0.01 U/mg) was decreased to 65% of WT-P control (0.83 ± 0.02 U/mg) and WT-P-ack (0.84 ± 0.02 U/mg); (ii) ACK activity was barely changed in WT-P-ack (9.51 ± 0.21 U/mg) compared to WT-P (9.19 ± 0.92 U/mg) and WT-P-pta (8.69 ± 0.36 U/mg). The *pta* asRNAs performed better than *ack* asRNAs in repression. The strain expressing *pta* asRNAs was further characterized.

There are a few possible reasons that could explain the observed difference in repression exerted by *pta* and *ack* asRNAs. AsRNA repression follows a threshold linear response (Georg and Hess, 2011), which suggests that RNA repression only occurs when the abundance of asRNAs is higher than a certain threshold and then with a continuing increase in asRNA abundance, repression will gradually increase. It is possible that WT-P-pta and WT-P-ack strains have different thresholds depending on the abundance of *pta* or *ack* transcripts, and the abundance of asRNAs may vary due to differential vulnerability to ribonucleases. In addition, the RNA structure is important to the physical binding between asRNAs and target transcripts that is necessary for RNA repression. RNA structure prediction (Gruber et al., 2008) found that the *ack* target region is more likely to form a secondary structure (**Supplementary Figure S1**), which may influence asRNA binding. Although we did not evaluate the extent that these factors could affect RNA repression, our study indicated the variability of asRNA repression and suggested the potential importance of asRNA design and promoter activity in maximizing RNA repression.

### Metabolic Changes in Knock-Down Strains

**FIGURE 2:**
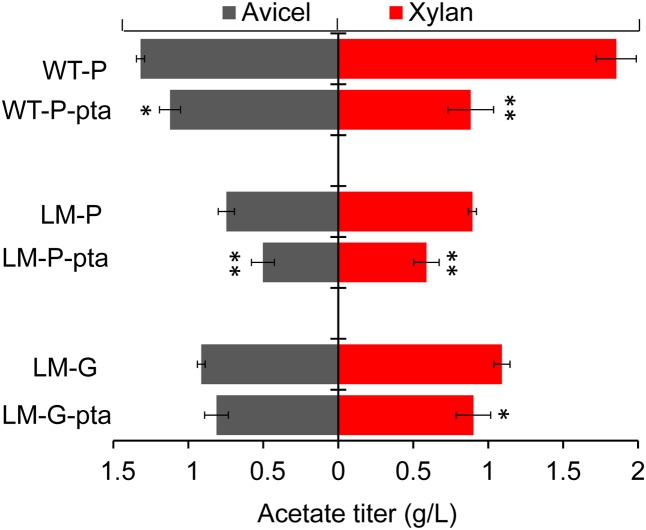
Comparison of acetate titers produced on 10 g/L Avicel cellulose **(Left)** and 10 g/L xylan **(Right)**. Strain names are labeled on the left, where WT-P and LM-P represent empty vector transformants of wildtype and lactate production-deficient mutant LM respectively, LM-G represents the chromosomal integrant with an empty repression module, all others carry a *pta* repression module either in plasmid (WT-P-pta and LM-P-pta) or chromosome (LM-G-pta). Error bar represents the standard deviation of three biological replicates. The asterisk (^∗^) indicates statistically significant differences between the engineered strain and its corresponding control (student’s *t*-test, ^∗^*P* < 0.05, ^∗∗^*P* < 0.01).

We measured the titers of three major metabolites (lactate, acetate, and ethanol) at the end of batch fermentations to determine if acetate production was decreased. With 10 g/L cellulose, the WT-P-pta strain produced lactate, acetate, and ethanol in a molar ratio of 0.93:1.37:1, compared to 1.75:1.49:1 in WT-P control (Supplementary Table S2). The acetate titer in WT-P-pta was decreased about 15% relative to the titer of WT-P (**Figure 2**). Interestingly, the lactate titer was decreased more than 50% in WT-P-pta, but ethanol production was not significantly changed (Supplementary Table S2). When both strains were grown on 10 g/L xylan, acetate became the major product, which is consistent with previous studies (Li et al., 2012); strikingly, WT-P-pta substantially reduced acetate titer to less than 48% of WT-P (**Figure 2**), corresponding to a molar ratio of acetate to ethanol of 4.11:1 in WT-P-pta versus 6:1 in WT-P (Supplementary Table S2). Notably, the *pta* asRNAs expressed in WT performed very well in reducing acetate production even though carbon sources greatly change metabolic profiles. The unexpected decrease in lactate titer on cellulose, as a side effect of manipulating acetate-producing genes, suggests a more sophisticated metabolic regulatory network in this strain, which was also supported by the reported decrease in acetate production in the lactate production-deficient LM mutant (Li et al., 2012). However, in *C. thermocellum* the Δ*pta* knockout mutant dramatically increased lactate titer (Argyros et al., 2011), which is in contrast to the accompanying decrease in lactate titer in the *pta* knockdown mutant of *C. cellulolyticum*. It seems like *Clostridium* strains employ different strategies to coordinate metabolic networks. In addition, despite the operability of *pta* disruption in some strains, the resulting effectiveness in acetate formation varies a lot. For example, *pta* deletion reduced acetate by just 14% in *Clostridium tyrobutyricum* (Zhu et al., 2005), but completely eliminated it in *C. thermocellum* (Argyros et al., 2011) and *Thermoanaerobacterium saccharolyticum* (Shaw et al., 2008).

Next, we transformed pRNAi-pta into the lactate production-deficient LM mutant to generate an LM-P-pta strain that should be deficient in both lactate and acetate production. A control strain, LM-P, was created in parallel by transforming pRNAi that cannot express any specific asRNAs. Metabolic profiling revealed that on 10 g/L cellulose, the LM-P control produced lactate, acetate and ethanol with a molar ratio of 0.04:0.55:1 (Supplementary Table S2); LM-P-pta made negligible lactate, a 33% decrease in acetate titer (**Figure 2**) and an 86% increase in ethanol titer, resulting in a molar ratio of 0.001:0.20:1 (lactate: acetate: ethanol). On 10 g/L xylan, the titers of lactate and acetate in LM-P-pta were decreased about 82 and 34% (**Figure 2**), respectively, and ethanol titer was slightly increased, corresponding to a molar ratio of 0.06:0.83:1 in LM-P and 0.01:0.51:1 in LM-P-pta (Supplementary Table S2). Hence, with the customized *pta* asRNAs expressed in transformants, we successfully manipulated both lactate and acetate producing pathways simultaneously.

Comparing the molar ratio of the three major end products (lactate, acetate and ethanol) in the control and asRNA expressing strains, we found that both WT-P-pta and LM-P-pta produced an equal molar amount of ethanol by generating less lactate and acetate, regardless of carbon source (Supplementary Table S2). In another word, these repression strains recovered more carbon in the form of ethanol. For instance, when LM-P-pta was grown on cellulose, 83% of the carbons used to produce the three major metabolites were accounted for in the ethanol, 21% higher than the corresponding control (Supplementary Table S2). This demonstrates a successful manipulation of essential metabolic genes to divert carbon flux toward ethanol production.

### Chromosomal Integration and Functional Analyses

**FIGURE 3:**
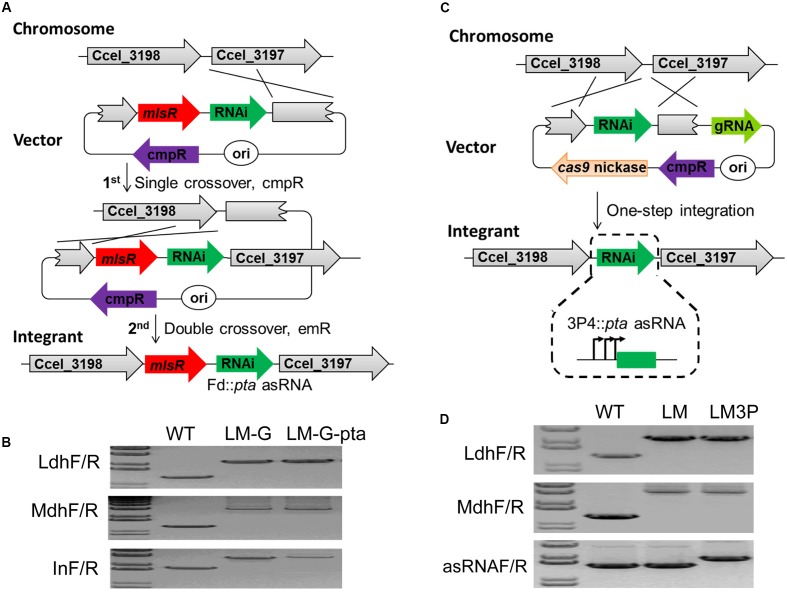
Chromosomal integration of functional modules via double-crossover recombination **(A,B)** and the Cas9 nickase genome editing tool **(C,D)**. The integration site was located in the intergenic region between Ccel_3198 and Ccel_3197. **(A)** Generation of stable double-crossover clones, LM-G and LM-G-pta, using pLyc045 and pLyc046, respectively. The first step was to screen thiamphenicol-resistant single-crossover clones generated by plasmid integration. The second step was to select erythromycin-resistant double-crossover clones as a result of plasmid excision. Finally, modified genomic loci in candidate clones were verified by PCR with specific primers, LdhF/R for Δ*ldh* identification, MdhF/R for Δ*mdh* identification and InF/R for module integration **(B)**. **(C)** Generation of stable chromosomal integrants, LM3P and LM3PS, by the Cas9 nickase genome editing tool. By transforming pCas9n-3198D-donor into the LM mutant, integrants were generated within a single step. **(D)** Modified genome loci in all integrants were then verified by PCR with specific primers, asRNAF/R for RNAi module integration. LM is a double mutant (Δ*ldh Δmdh*); LM-G-pta and LM3P are triple mutants (Δ*ldh Δmdh Δpta*).

In light of the effectiveness of *pta* asRNAs in reducing acetate production, we attempted to integrate the asRNA-expressing module into the genome of the LM mutant in such a way that resulting integrants can work stably and desirably without using antibiotics. To do so, step-wise double-crossover recombination was initially applied before the use of Cas9 technology in Clostridia (Heap et al., 2012) (**Figure 3A**). The integration site was immediately downstream of the sole bifunctional acetaldehyde-CoA/alcohol dehydrogenase-encoding gene (*adhE*) in *C. cellulolyticum* (Ccel_3198). The specific integration can generate an artificial bicistronic operon containing the open reading frames of *adhE* and *mlsR* under the control of the native *adhE* promoter, consequently enabling counter selection of double-crossover events with erythromycin. During the screening of double-crossover events, pure LM-G-pta integrants (where G indicates genome/chromosome-based expression) were isolated after two rounds of plate streaking. Targeted integration in LM-G-pta and LM-G controls was verified by PCR amplification (**Figure 3B**) and amplicon sequencing.

The functionality of the integrated P4::*pta* asRNA module was evaluated by measuring PTA activity and fermentation products. In comparison, the crude extract of cellobiose-grown LM-G-pta integrant presented a lower PTA activity (0.92 ± 0.04 U/mg) that was 89% of LM-G control (1.13 ± 0.06 U/mg), indicating the integrated module was still functional but did not perform as well as the plasmid-based repression in WT-P-pta (**Figure 1C**). Metabolically, the acetate titer in LM-G-pta did not significantly reduce on 10 g/L cellulose but dropped 17% on 10 g/L xylan (**Figure 2**). The overall molar ratios (lactate: acetate: ethanol) were changed from 0.05:0.59:1 in LM-G to 0.01:0.31:1 in LM-G-pta when cultured on cellulose, and correspondingly from 0.09:1.13:1 to 0.04:0.84:1 on xylan (Supplementary Table S2). In general, the integrant was not comparable with the aforementioned transformant in repressing enzymatic and metabolic activities. Previous studies have found that small RNA repression follows a threshold-linear model distinct from protein-mediated repression (Levine et al., 2008; Georg and Hess, 2011). In this case, with a fixed transcription rate of chromosomal *pta* gene, switching from plasmid-based to chromosome-based asRNA expression presumably reduces asRNA dosage, which would weaken the repression of acetate formation.

### Evaluation of the Gene-Dosage Effect between Transformants and Chromosomal Integrants

**FIGURE 4:**
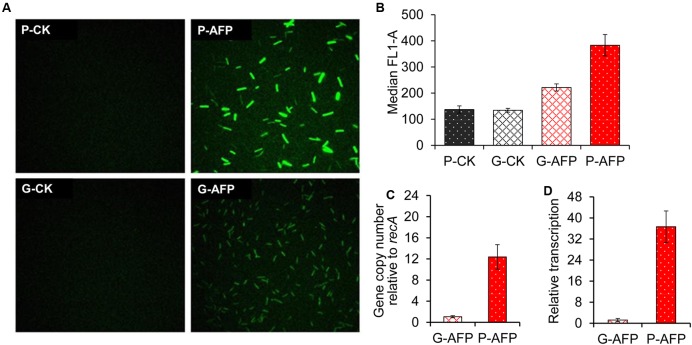
Expression of anaerobic fluorescent protein in the P-AFP transformant and the G-AFP integrant. **(A)** Fluorescence microscopy of cellobiose-grown cells at the mid-log phase. P-CK and G-CK were corresponding controls of P-AFP and G-AFP that carry the reporter gene in plasmid and chromosome, respectively. **(B)** Quantification of fluorescent signal intensity with flow cytometry. **(C)** Relative *afp* gene copy number in both G-AFP and P-AFP by reference to the single chromosomal *recA* gene. **(D)** qRT-PCR comparison of *afp* transcript levels between G-AFP and P-AFP, with normalization to *recA* calibrator. Error bar represents the standard deviation of three biological replicates.

To determine if chromosomal integration mitigates gene activity and how strong the effect is, an *afp* gene encoding anaerobic fluorescent protein was introduced into either the plasmid (P-AFP) or the genome (G-AFP) and then their respective activities were visualized and compared. As expected, P-AFP presented much stronger signal intensity than G-AFP under fluorescent microscopy (**Figure 4A**). Then, quantification of the fluorescence signal by flow cytometry revealed that the signal intensity of P-AFP was 1.73-fold higher than that of G-AFP (**Figure 4B**); when compared to corresponding negative controls (P-CK and G-CK), P-AFP and G-AFP generated 2.79-fold and 1.65-fold greater fluorescent intensity, respectively. The lower signal intensity of G-AFP directly reflects a lower AFP activity and presumably indicates a lower amount of *afp* transcripts produced in G-AFP. Quantitative real-time PCR (qRT-PCR) analysis supported this assumption, showing that relative to G-AFP, P-AFP harbored a 12-fold higher abundance in *afp* gene copy number (**Figure 4C**) and a 36-fold higher abundance in *afp* transcript (**Figure 4D**). These results together indicate that chromosomal integration substantially altered the dosage of gene expression and then diminished gene activity. High-copy number pJIR750 derivatives, including pLyc17 used here to generate P-AFP transformants, have also been reported in *Clostridium perfringens*, which carried as many as 18 copies (Cheung et al., 2009).

### Improved Repression of Acetic Acid Production by a Tandem Repetitive Promoter

To overcome the weakened asRNA repression observed with chromosomal integration, we attempted to improve promoter activity to increase asRNA production by introducing a tandem promoter which consists of three P4 repeats, named 3P4. P4 is a 36-bp synthetic promoter with an activity comparable to the strong ferredoxin (Fd) promoter in *C. cellulolyticum* (Xu et al., 2015). Then, a 3P4::pta asRNA module was constructed and integrated into the LM genome at the same locus by a Cas9 nickase-based editing tool (Xu et al., 2015) (**Figure 3C**), generating a LM3P integrant (Δ*ldh*Δ*mdh*Δ*pta*). After transforming the single all-in-one vector, we randomly picked three antibiotic-resistant transformants, all of which were verified to be correct integrants by PCR amplification (**Figure 3D**) and amplicon sequencing. Methodologically, although both double-crossover recombination and Cas9 nickase-triggered homologous recombination have the ability to integrate asRNA modules as shown in **Figure 3**, the latter presents multiple advantages, such as markerless editing, one-step generation and high editing efficiency (Xu et al., 2015).

**FIGURE 5:**
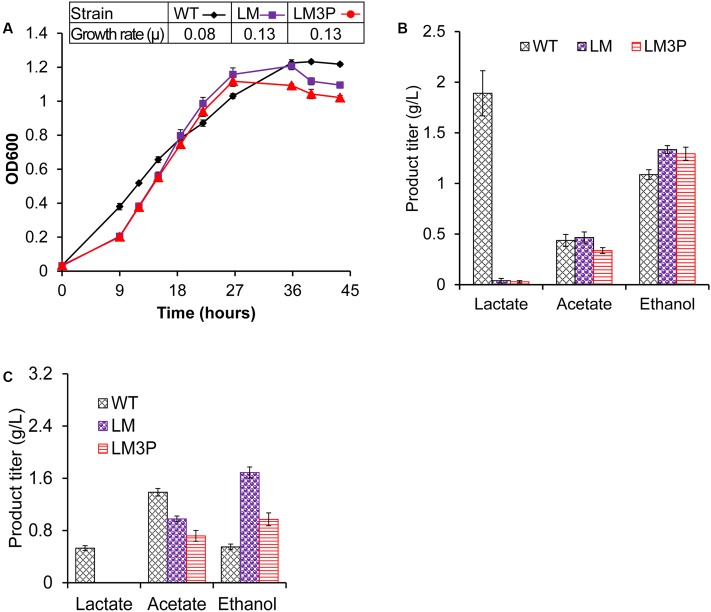
Comparison of growth profiling and product measurement between wildtype (WT), lactate production-deficient mutant (LM), and chromosomal integrant of LM with an enhanced 3P4::pta asRNA module (LM3P). **(A)** Cell growth was profiled with an insert table showing the growth rate of each strain under the tested condition. Product titers in the end-point fermentation broth were measured at the end fermentation with 5 g/L cellobiose **(B)** or 10 g/L cellulose **(C)**. Error bar represents the standard deviation of three biological replicates.

The chromosomal asRNA module is supposed to repress the *pta* gene independent of plasmid-borne elements and antibiotic utilization. When grown on 5 g/L cellobiose, both LM and LM3P presented similar biomass yields and growth rates (μ = 0.13 h^-1^) (**Figure 5A**), almost double the growth rate of WT (μ = 0.08 h^-1^) while LM3P’s acetate titer decreased by 28% relative to LM (**Figure 5B**). On 10 g/L cellulose, LM3P produced similar amount of cell biomass as LM but significantly reduced the acetate titer by 27% (**Figure 5C**), suggesting much stronger gene repression triggered by the integrated 3P4::pta asRNA module than by the previous Fd::pta asRNA module in LM-G-pta. The enhanced repression in LM3P even got close to the plasmid-based repression in LM-P-pta. In addition, LM3P produced less ethanol than LM on cellulose (**Figure 5C**). This reduction was not due to a negative effect of chromosomal integration on the neighboring *adhE* gene since the alcohol dehydrogenase activity responsible for acetaldehyde reduction was not reduced but instead increased in the crude extracts of LM3P (Supplementary Table S3). It is possible that cellular redox balancing strategies changed the reducing power for ethanol production and the carbon flow for acetate production (Desvaux et al., 2000).

Although CRSIPRi also can allow stable manipulation by integrating both non-catalytic CRSIPR endonuclease and gRNA cassettes, it is challenging to introduce the large-size endonuclease genes into microbial chromosomes (Kung et al., 2013), and whether the permanent expression of foreign CRISPRi will interfere with host CRISPR machinery or host physiology needs to be evaluated in specific hosts (Barrangou, 2015; Luo et al., 2016). Our study used transient plasmid-dependent Cas9n to deliver stand-alone asRNA modules into the genome, able to circumvent aforementioned challenges. It would also be interesting to systematically compare the repression efficiency and specificity of asRNAs and CRISPRi in bacterial hosts.

## Conclusion

Antisense RNA-mediated repression worked well in both the *C. cellulolyticum* wildtype and LM mutant to repress *pta* expression thereby reducing acetate production in these strains. Combined utilization of gene repression and Cas9 nickase genome editing realized a one-step markerless integration of an upgraded antisense RNA-expressing module into the chromosome, genetically allowing stable manipulation of essential genes and providing a technical demonstration of the unmatched editing simplicity and efficiency of this system over double-crossover recombination. The tandem promoter strategy dramatically improved repression of acetate formation in the integrants. This combinatorial strategy significantly expanded our ability to manipulate more diverse genes for functional characterization and strain engineering.

## Author Contributions

TX and YL designed the study and performed the experiments. TX drafted the manuscript. JVN helped data visualization and revised the manuscript. ZH and JZ participated in the conception of the study, helped data interpretation and revised the manuscript. All authors read and approved the final manuscript.

## Conflict of Interest Statement

The authors declare that the research was conducted in the absence of any commercial or financial relationships that could be construed as a potential conflict of interest.

## Supplementary Material

FIGURE S1 RNA structures of targeted transcript regions of *pta* **(A,B)** and *ack* **(C,D)** predicted by RNAfold web server. The color represents base-pair probabilities. **(A,C)** Secondary structure with minimal free energy; **(B,D)** centroid secondary structure.
