# Supplementary figures and images for: Cas9 Nickase-Assisted RNA Repression Enables Stable and Efficient Manipulation of Essential Metabolic Genes in Clostridium cellulolyticum
Source: Front Microbiol. 2017 Sep 7;8:1744. doi: 10.3389/fmicb.2017.01744 (PMC5594222; doi:10.3389/fmicb.2017.01744)

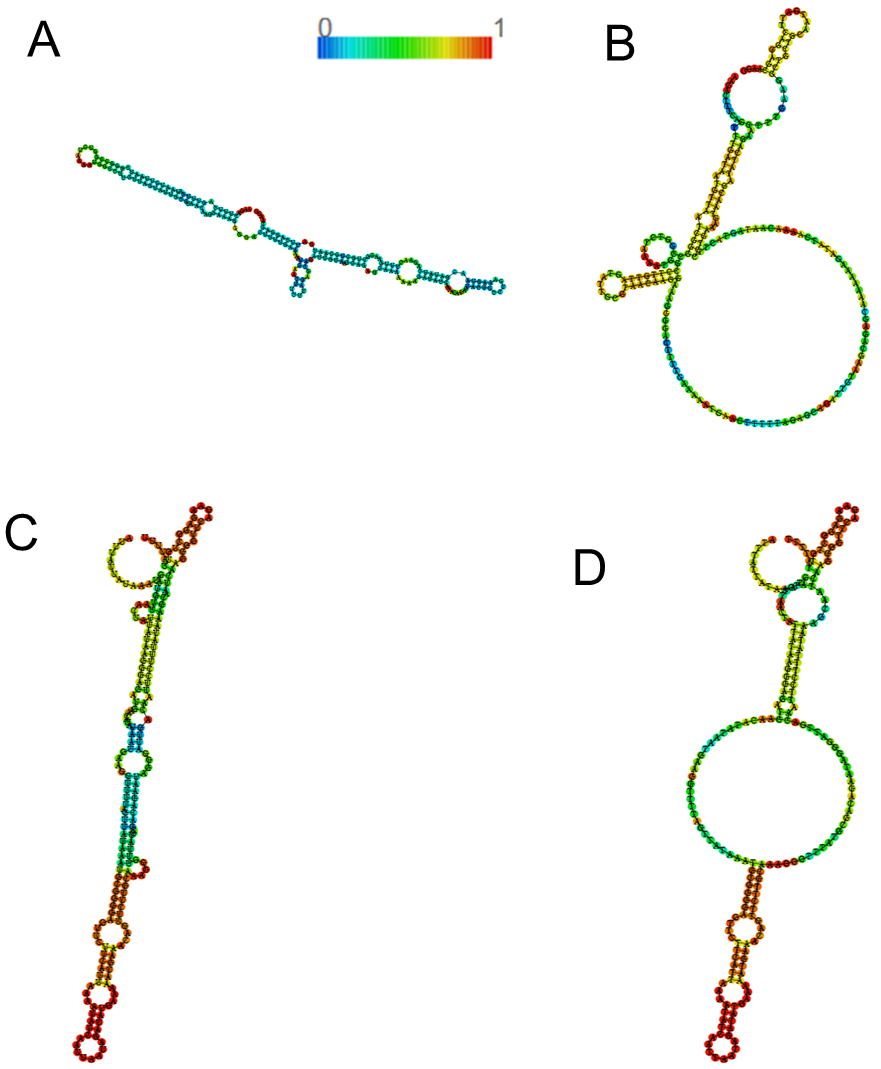

Supplement: FIGURE S1 — RNA structures of targeted transcript regions of pta (A,B) and ack (C,D) predicted by RNAfold web server. The color represents base-pair probabilities. (A,C) Secondary structure with minimal free energy; (B,D) centroid secondary structure. [file Image_1.TIF]
