# Supplementary material for: Cas9 Nickase-Assisted RNA Repression Enables Stable and Efficient Manipulation of Essential Metabolic Genes in Clostridium cellulolyticum
Source: Front Microbiol. 2017 Sep 7;8:1744. doi: 10.3389/fmicb.2017.01744 (PMC5594222; doi:10.3389/fmicb.2017.01744)
Supplement: Supplementary file 2 [file Table_1.PDF]

Table S1 List of primers used in this study.

Primer	**sequence**	**Note**
ptaF	aaccgagctcggtacccgggCCTTGCTCCTGAATCATTG	Amplify partial pta region
ptaR	gcgatcgttcgactctagagAAGACTTTCAGTTTGATAATGTTAATC	Amplify partial pta region
ackF	aaccgagctcggtacccgggAAAATAACAGCCTCTTCTGAAC	Amplify partial ack region
ackR	gcgatcgttcgactctagagATTTATTATAAATGATGTTAACTATTATAAGG	Amplify partial ack region
pRNAiF	GTAATCATGGTCATAGCTGTTTCCTG	Amplify pRNAi backbone
pRNAiR	CAGTTGCGCAGCCTGAATGG	Amplify pRNAi backbone
3198upF	acagctatgaccatgattacGGCCGGCCATTCCATCACCT	Amplify upper homologous region
3198upR	tccttgtacaTTACTTGCCGTAGTAAACTTTCAGGTACATGTC	Amplify upper homologous region
mlsRF	cggcaagtaaTGTACAAGGAGGTTTACAATG	Amplify promoter-less mlsR
mlsRR	gcgcgaattcCCATGGTTACTTATTAAATAATTTATAGC	Amplify promoter-less mlsR
asRNAF	gtaaccatggGAATTCGCGCCCCGGATCGA	Amplify asRNA cassette
asRNAR	tttcctcgagTTTTATAGGGCGTGTTTGTGGCTTAGAG	Amplify asRNA cassette
3198downF	ccctataaaaCTCGAGGAAACATTTGGTTC	Amplify lower homologous region
3198downR	ccattcaggctgcgcaactgAAGCTTTTTGCATTTTTTACTTC	Amplify lower homologous region
afpF	gtaaccatggGAATTCGAGCTCGGTACCCG	Amplify Fd::afp cassette
afpR	tttcctcgagTTTTATAGGGCGTGTTTGTGGC	Amplify Fd::afp cassette
ldhF	TATACTTTTGCACCCAGAATGTTTT	Identify Δldh mutants
ldhR	TGACTGATACGGGTTTTATCAATTT	Identify Δldh mutants
mdhF	GGGATTTTAATGGGTTTTAAAGTTG	Identify Δmdh mutants
mdhR	TCCAGGTGAATAAGCTAAAGAAAGA	Identify Δmdh mutants
InF	CAGGCAACTAAGAACATTTTTGAAT	Identify asRNA integrants
InR	CCTCCAGAGTACCAGTTAATTCTGA	Identify asRNA integrants
3P4F	gcaagtaagaaacatttggcGCTTCACGTGATCCATGGCA	Amplify 3P4 promoter cluster
3P4R	GATTCAGGAGCAAGGATCCATGGAAGCTTCAAC	Amplify 3P4 promoter cluster
3P4ptaF	CCATGGATCCTTGCTCCTGAATCATTGCAGC	Amplify partial pta region for 3P4::pta asRNA construct
3P4ptaR	gcccggtacaaaaccggaacAAGACTTTCAGTTTGATAATGTTAATCGG	Amplify partial pta region for 3P4::pta asRNA construct
3198LF	gaattttattatggtacccgggGTGCTGAGGCAATGAGGGAT	Amplify 1-kb right arm of 3198 donor
3198LR	ggaaCCATGGccaaatgtttcTTACTTGCCGTAG	Amplify 1-kb right arm of 3198 donor
3198RF	GGCAAGTAAgaaacatttggCCATGGttccggttttgtaccgggcacgtgg	Amplify 1.4-kb left arm of 3198 donor
3198RR	catcaatttgttgcaacgagACGGCATTCTTTGTAGCCCA	Amplify 1.4-kb left arm of 3198 donor
RTafpF	ATCAGGACGCCCGTTTTCTT	qRTPCR
RTafpR	ACCGGTGTGATGGACAACTC	qRTPCR
RTrecAF	GCAAAGAAACTTGGGGTTGA	qRTPCR
RTrecAR	TGAGACATCAGCCTTGCTTG	qRTPCR
