# Supplementary material for: Cas9 Nickase-Assisted RNA Repression Enables Stable and Efficient Manipulation of Essential Metabolic Genes in Clostridium cellulolyticum
Source: Front Microbiol. 2017 Sep 7;8:1744. doi: 10.3389/fmicb.2017.01744 (PMC5594222; doi:10.3389/fmicb.2017.01744)
Supplement: Supplementary file 3 [file Table_2.PDF]

Table S2 Measurement and comparison of product titers and molar ratios in the fermentation broth of *C. cellulolyticum* strains grown on cellulose and xylan.

Carbon	Strain	Lactate [a]	Acetate	Ethanol	Molar ratio of lactate : acetate : ethanol [d]	Carbon in ethanol (%) [e]
	WT-P-pta/	1.159±0.154 [b]/	1.122±0.071/	0.639±0.021/	0.93 : 1.37 : 1/	26.56/
	WT-P	2.358±0.199 (0.49)**[c]	1.319±0.027 (0.85)*	0.691±0.036	1.75 : 1.49 : 1	19.55
10 g/L	LM-P-pta/	0.002±0.008/	0.503±0.077/	1.963±0.203/	0.001 : 0.2 : 1/	83.23/
Cellulose	LM-P	0.078±0.002 (0.03)**	0.747±0.040 (0.67)**	1.055±0.162 (1.86)**	0.04 : 0.55 : 1	62.11
	LM-G-pta/	0.024±0.004/	0.812±0.079/	2.074±0.308/	0.01 : 0.31 : 1/	75.47/
	LM-G	0.110±0.011 (0.22)**	0.914±0.026	1.211±0.283 (1.71)**	0.05 : 0.59 : 1	60.06
	WT-P-pta/	0.251±0.132/	0.884±0.209/	0.168±0.038/	0.76 : 4.11 : 1/	16.00/
	WT-P	0.357±0.085	1.854±0.193 (0.48)**	0.241±0.042	0.76 : 6 : 1	12.29
10 g/L	LM-P-pta/	0.019±0.004/	0.588±0.085/	0.906±0.144/	0.01 : 0.51 : 1/	65.57/
Xylan	LM-P	0.104±0.011 (0.18)**	0.894±0.027(0.66)**	0.844±0.145 (1.07)**	0.06 : 0.83 : 1	52.08
	LM-G-pta/	0.059±0.034/	0.902±0.115/	0.835±0.172/	0.04 : 0.84 : 1/	52.63/
	LM-G	0.126±0.019 (0.47)*	1.091±0.054 (0.83)*	0.754±0.091	0.09 : 1.13 : 1	44.15

[a]Product titers (g/L) and molar ratios of the engineered strain and its control are divided by a forward slash.
[b]All data is presented as mean± standard deviation (n=3). Student's t test was applied to test the significance of a difference (*P<0.05;**P<0.01).
[c]The numbers in parentheses represent the titer ratio if the difference is statistically significant.
[d]The molar ratio is calculated by normalizing to ethanol.
[e]The percentage of ethanol-containing carbon in three major metabolites including lactate, acetate and ethanol.
