# Supplementary material for: Cas9 Nickase-Assisted RNA Repression Enables Stable and Efficient Manipulation of Essential Metabolic Genes in Clostridium cellulolyticum
Source: Front Microbiol. 2017 Sep 7;8:1744. doi: 10.3389/fmicb.2017.01744 (PMC5594222; doi:10.3389/fmicb.2017.01744)
Supplement: Supplementary file 4 [file Table_3.PDF]

Table S3 Measurement of alcohol dehydrogenase activity in crude extracts.

Cellobiose-grown strain	ADH activity (U/mg protein)
LM-G	0.053±0.003
LM-G-pta	0.062±0.003
WT	0.008±0.001
LM	0.037±0.001
LM3P	0.052±0.001

Values are presented as mean± standard deviation.
